# Supplementary figures and images for: Drosophila immune priming to Enterococcus faecalis relies on immune tolerance rather than resistance
Source: PLoS Pathog. 2023 Aug 11;19(8):e1011567. doi: 10.1371/journal.ppat.1011567 (PMC10446173; doi:10.1371/journal.ppat.1011567)

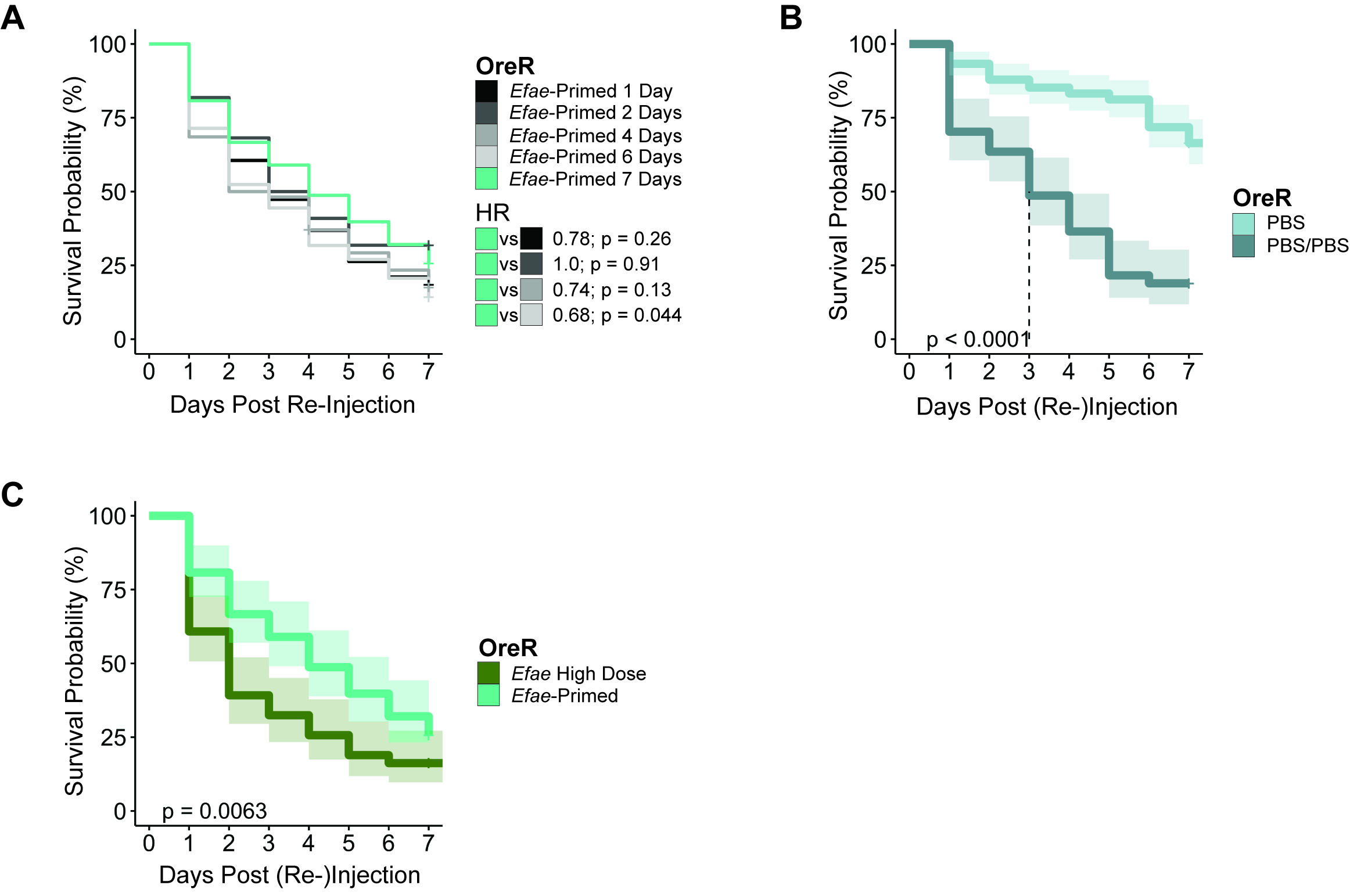

Supplement: S1 Fig — A). Survival is similar in flies allowed to prime with a low-dose of E. faecalis (~3,000 CFU/fly) for varying amounts of time before re-infection with a high dose of E. faecalis (~30,000 CFU/fly) (n: 1 Day = 38, 2 Days = 22, 4 Days = 54, 6 Days = 63, 7 Days = 78). B). There is a significant difference in survival (log-rank sum test, p<0.0001) in OreR flies injected once with PBS (PBS, n = 149) or twice with PBS with seven days of rest between repeated injections (PBS/PBS, n = 74). Dotted lines indicate median survival time; shaded regions indicate 95% confidence intervals. C). There is a significant difference in survival (log-rank sum test, p = 0.0063) in OreR flies injected once with a high dose of E. faecalis (Efae High, ~30,000 CFU/fly, n = 74) versus primed with a low dose of E. faecalis for seven days and then re-infected with a high dose of E. faecalis (Efae-Primed, n = 78). Data are the same as Fig 1B and 1C, replotted for comparison. (TIF) [file ppat.1011567.s001.tif]

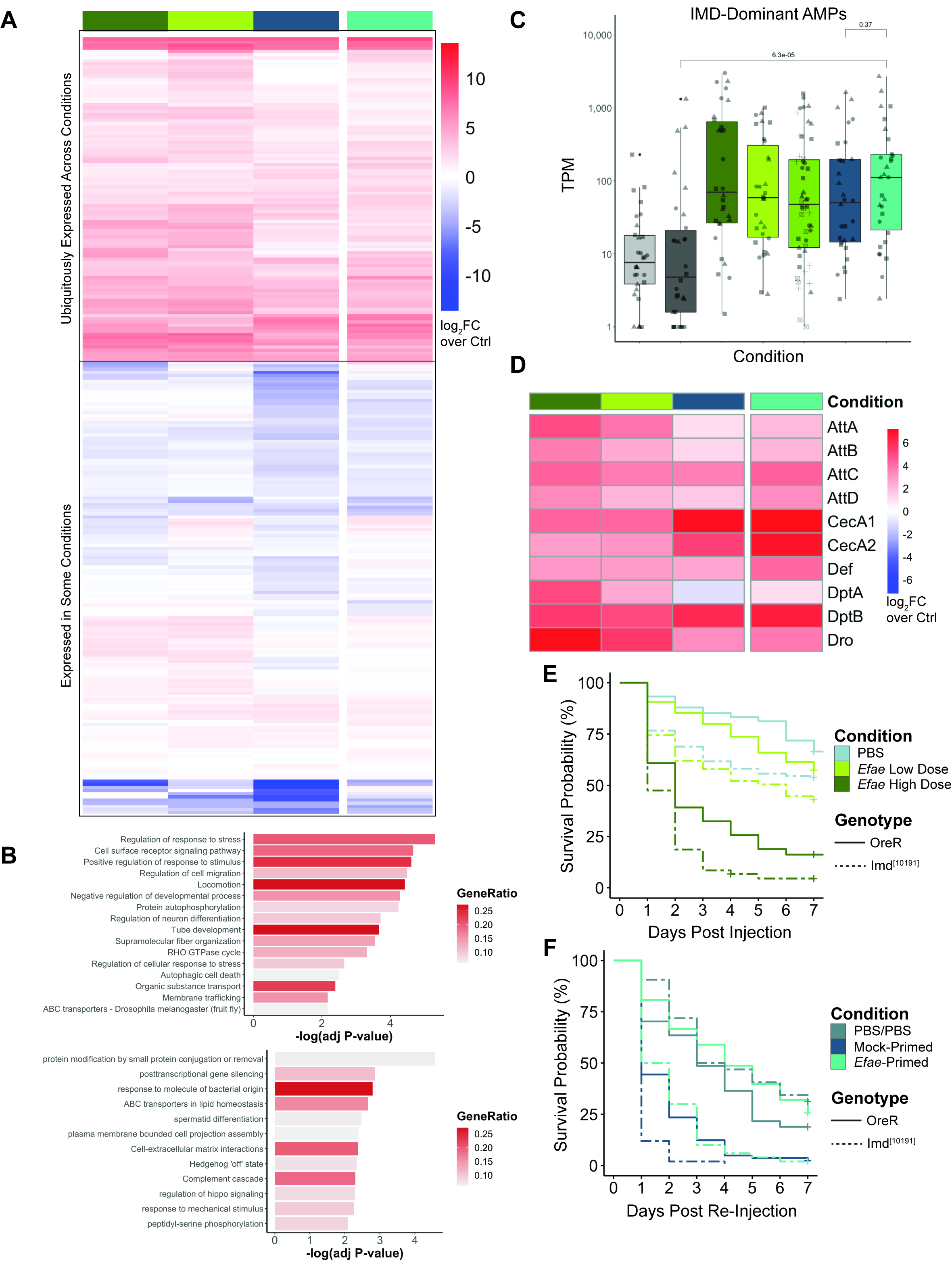

Supplement: S2 Fig — A). Heatmap of log2FC over non-injected controls of whole-body, core immune response genes from Troha, et al. 2018. Only a subset of the core genes were ubiquitously expressed across all conditions assayed for in this study. The differences are likey due to distinctions in time point and tissue. B). GO term enrichment from fat body Efae-Primed-specific [top] and Mock-Primed-specific [bottom], up-regulated genes. C). Expression in log10(TPM+1) of IMD-dominant AMPs. Biological replicates are designated by the shape of individual points. While there is a significant difference in IMD AMP expression in Efae-Primed fat bodies compared to their age-matched, non-injected controls (Wilcoxon test; p = 6.3E-05), there is not a significant difference in expression between Mock-Primed and Efae-Primed fat bodies (Wilcoxon test; p = 0.37). D). Heatmap of log2FC over non-injected controls of IMD-dominant AMPs across collected fat body samples E). Single-injection survival comparison between OreR and imd-mutant flies F). Double-injection survival comparison between OreR and imd-mutant flies. Data from D & E are the same as in Fig 3G and 3H, replotted for comparison. (TIF) [file ppat.1011567.s002.tif]

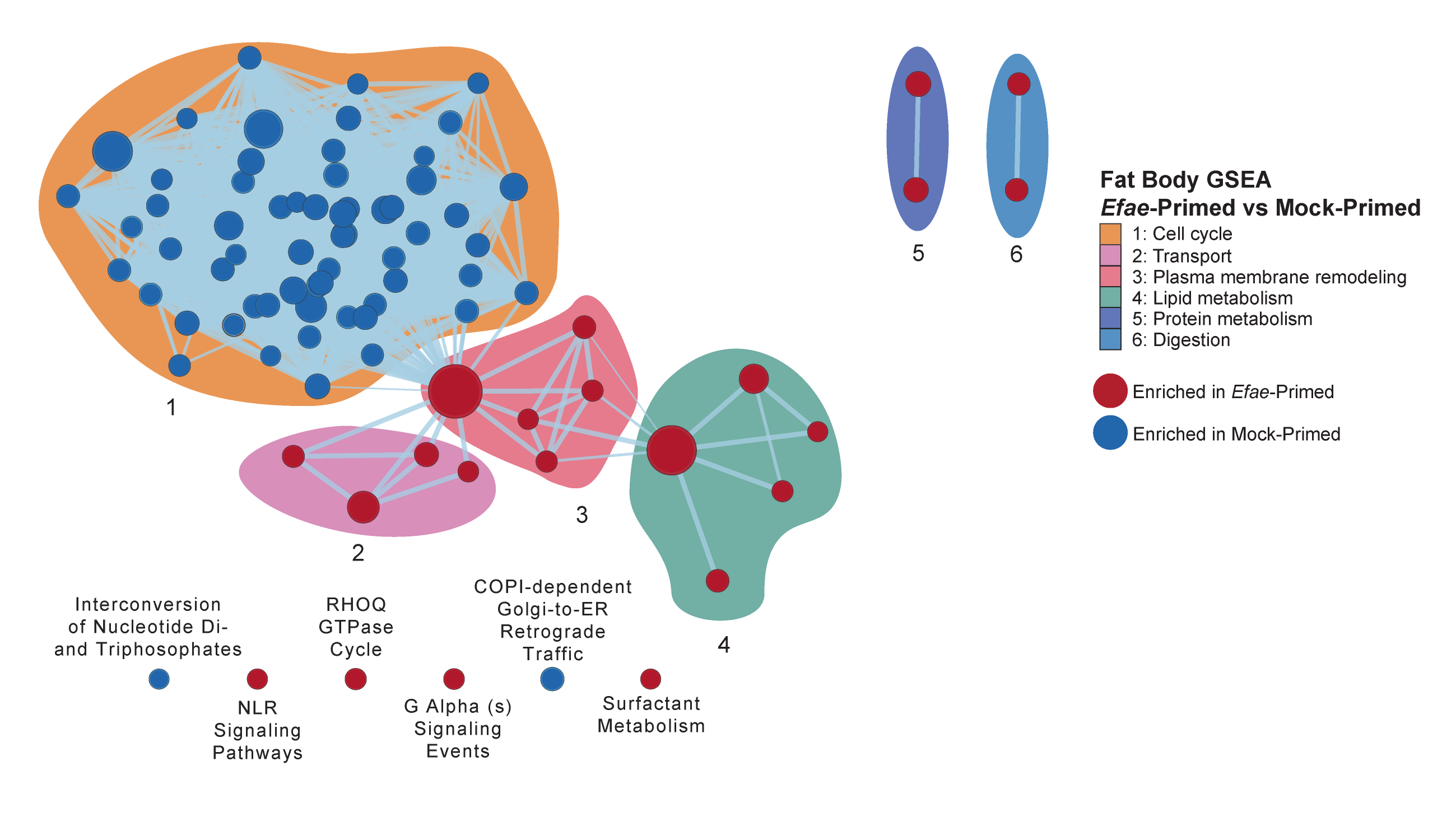

Supplement: S3 Fig — This visualization represents relationships between statistically significant terms (FDR < 0.05), manually curated with clusters that summarize the relationships between terms. Full results are found in S4 Table. (TIF) [file ppat.1011567.s003.tif]

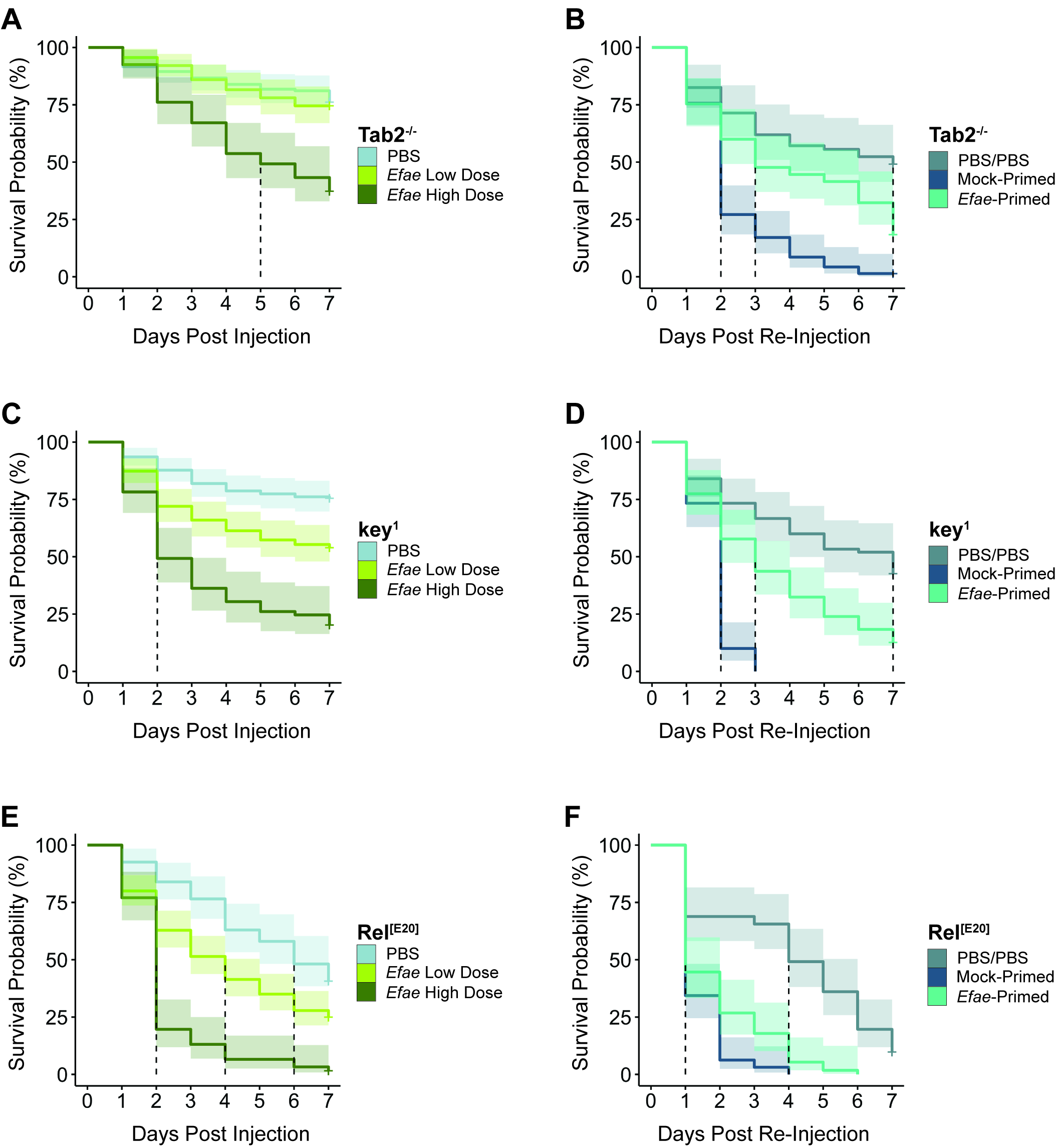

Supplement: S4 Fig — A). Survival of single-injected Tab2 mutant flies versus PBS control (PBS: n = 143, Efae Low Dose: n = 114, Efae High Dose: n = 67). Dotted line indicates median survival time. Shaded area indicates 95% confidence interval. Low Dose vs PBS: HR = 1.1, p = 0.76; High Dose vs PBS: HR = 3.4, p = 5.6E-08; pairwise comparisons are calculated using a Cox proportional hazard model with hazard ratios and Wald statistic values reported for experimental conditions versus their PBS negative control; significance values are adjusted for multiple testing using a Benjamini-Hochberg method. B). Survival of primed Tab2 mutant flies versus double-injected, non-primed controls (PBS/PBS: n = 63, Mock-Primed: n = 70, Efae-Primed: n = 65). Efae-Primed vs PBS/PBS: HR = 2.0, p = 0.0016; Mock-Primed vs. PBS/PBS: HR = 4.6, p = 1.1E-10. C). Survival of key mutant flies injected with PBS (n = 155), Efae Low Dose (~3,000 CFU/fly, n = 148), and Efae High Dose (~30,000 CFU/fly, n = 69). Low Dose vs PBS: HR = 2.2, p = 7.5E-05; High Dose vs PBS: HR = 5.3, p = 2.0E-16 D). Survival of primed key mutant flies versus double-injected, non-primed controls (PBS/PBS: n = 75, Mock-Primed: n = 60, Efae-Primed: n = 71). Efae-Primed vs PBS/PBS: HR = 2.3, p = 3.6E-05; Mock-Primed vs. PBS/PBS: HR = 7.1, p = 3.5E-14. E). Survival of single-injected Rel mutant flies versus PBS control (PBS: n = 140, Efae Low Dose: n = 63, Efae High Dose: n = 60). Low Dose vs PBS: HR = 0.57, p = 0.0014; High Dose vs PBS: HR = 2.8, p = 7.2E-08. F). Survival of primed Rel mutant flies versus double-injected, non-primed controls (PBS/PBS: n = 55, Mock-Primed: n = 64, Efae-Primed: n = 56). Efae-Primed vs PBS/PBS: HR = 3.7, p = 3.1E-09; Mock-Primed vs. PBS/PBS: HR = 5.7, p = 1.2E-13. Like imd mutants, Rel, key, and Tab2 mutants lost the ability to fully prime against E. faecalis infections. The relative severity of the loss does depend on the mutant, possibly due in part to differences in genetic background, with Relish mutants showing [file ppat.1011567.s004.tif]

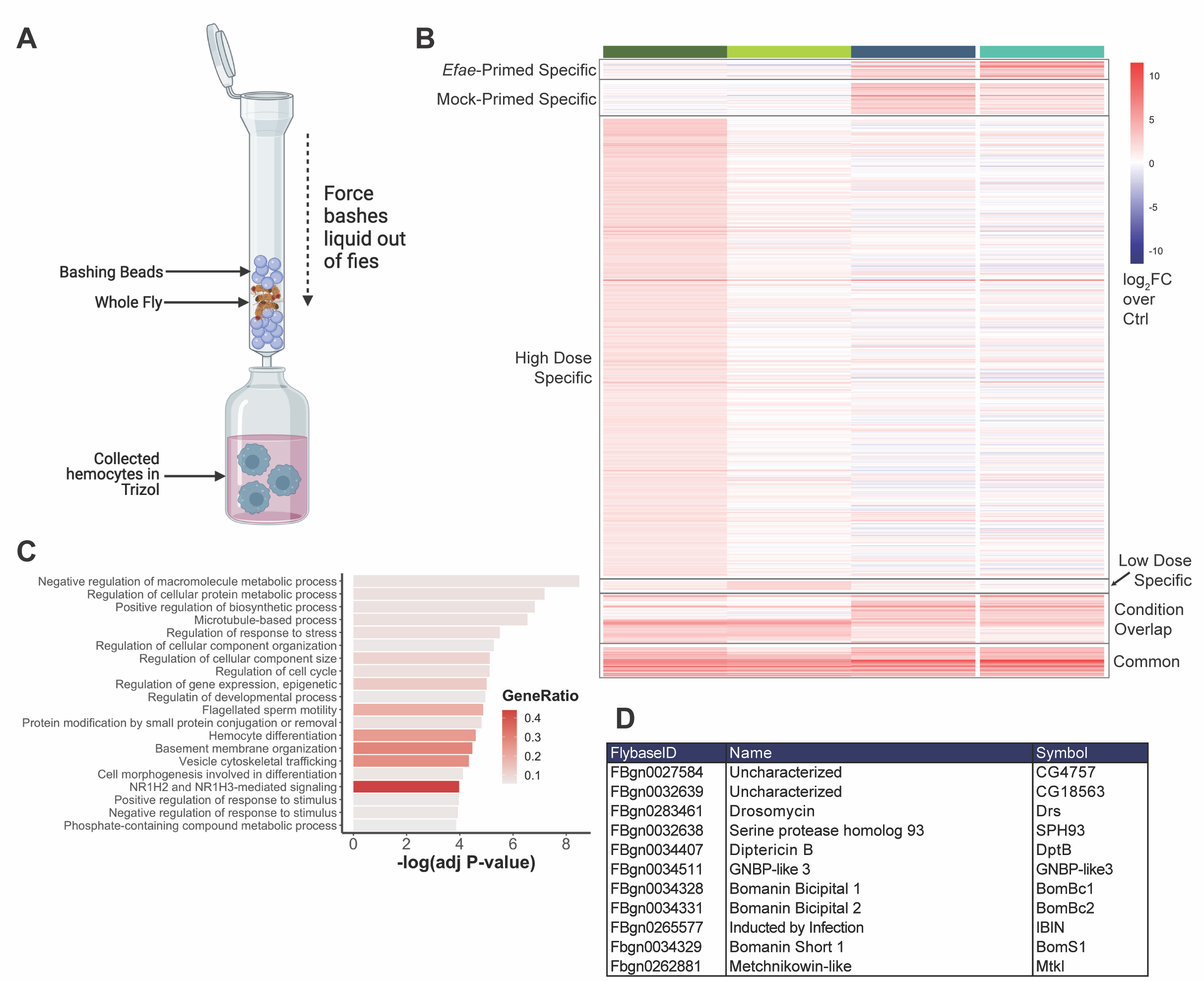

Supplement: S5 Fig — A). Schematic diagram of hemocyte RNA extraction. B). Significantly up-regulated genes as corresponding to conditions in Fig 4A (scale: log2FC over age-matched controls). C). GO term enrichment from hemocyte Efae Hi Dose-specific, up-regulated genes. D). Overlap of up-regulated core genes (4-condition overlap in Venn diagram) between hemocytes and fat bodies. Created with Biorender. (TIF) [file ppat.1011567.s005.tif]

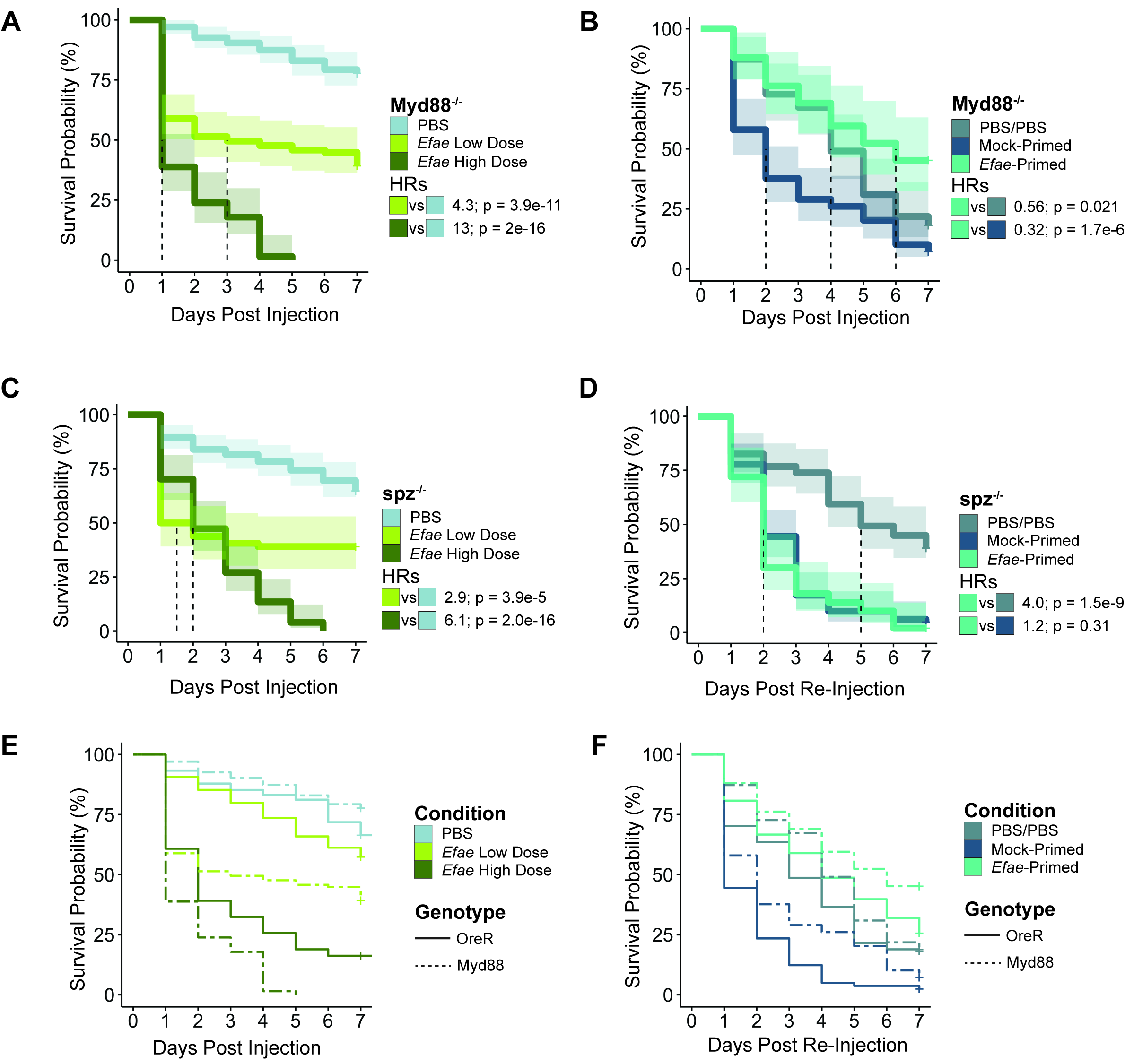

Supplement: S6 Fig — A). Survival of single-injected Myd88 mutant flies versus PBS control (PBS: n = 135, Efae Low Dose: n = 107, Efae High Dose: n = 67). Low Dose vs PBS: HR = 4.3 [2.8–6.6]; High Dose vs PBS: HR = 13 [8.8–22]. B). Survival of primed Myd88 mutant flies versus double-injected, non-primed controls (PBS/PBS: n = 60, Mock-Primed: n = 69, Efae-Primed: n = 60). Efae-Primed vs PBS/PBS: HR = 0.56 [0.34–0.92]; Mock-Primed vs. PBS/PBS: HR = 1.8 [1.2–2.7]. C). Survival of single-injected spz mutant flies versus PBS control (PBS: n = 64, Efae Low Dose: n = 65, Efae High Dose: n = 74). Low Dose vs PBS: HR = 2.9 [1.9–4.5]; High Dose vs PBS: HR = 6.1 [4.1–9.1]. D). Survival of primed spz mutant flies versus double-injected, non-primed controls (PBS/PBS: n = 69, Mock-Primed: n = 81, Efae-Primed: n = 50). Efae-Primed vs PBS/PBS: HR = 4.0 [2.6–6.1]; Mock-Primed vs. PBS/PBS: HR = 3.4 [2.3–5.1]. E). Single-injection survival comparison between OreR and Myd88-mutant flies. F). Double-injection survival comparison between OrR and Myd88-mutant flies. Data are the same as in A & B, replotted for comparison. (TIF) [file ppat.1011567.s006.tif]

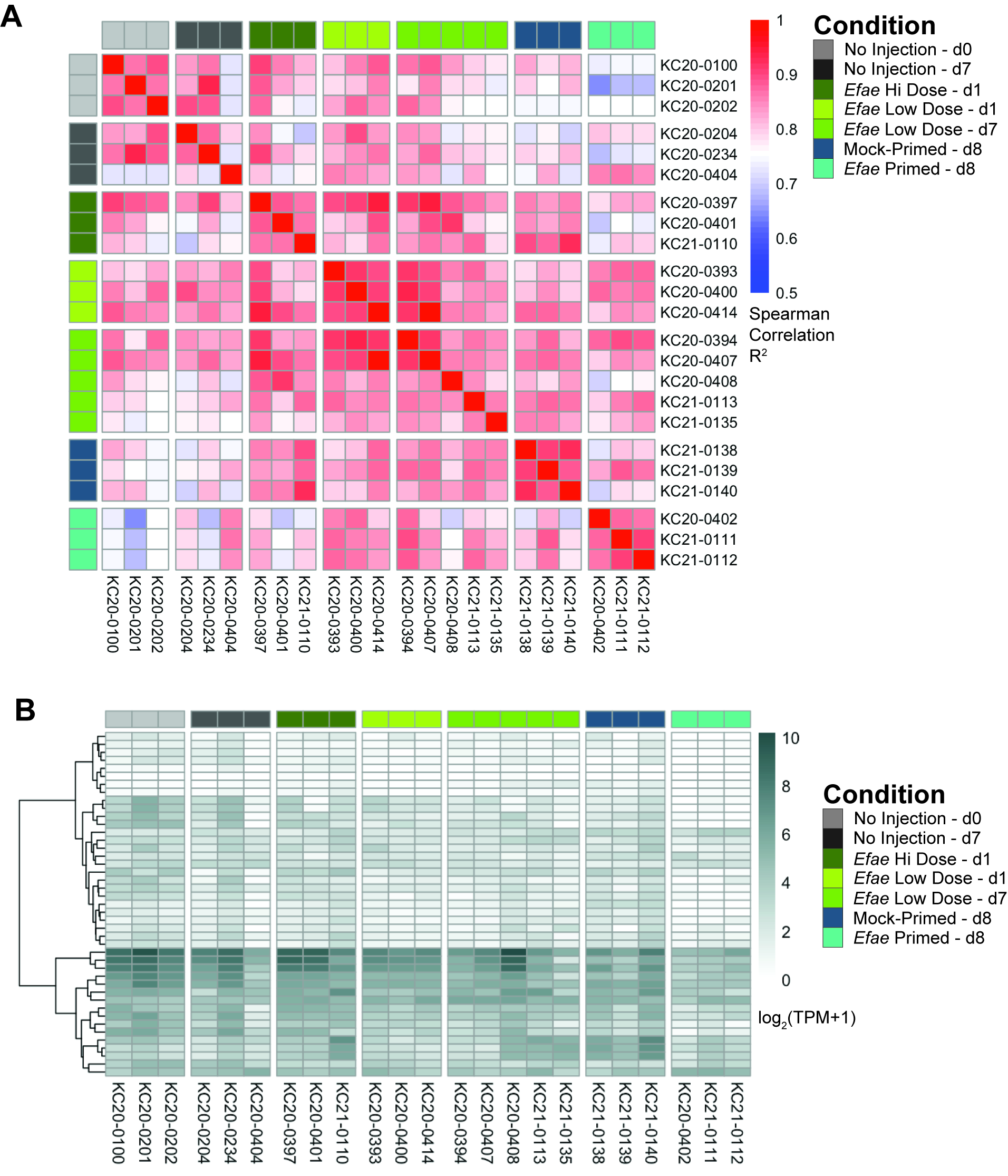

Supplement: S7 Fig — A). Spearman correlation heatmap of fat body RNA-seq libraries. Values are R2 spearman correlation values. B). Expression of sperm motility genes in fat body RNA-seq libraries. Values are log2(TPM+1). (TIF) [file ppat.1011567.s007.tif]

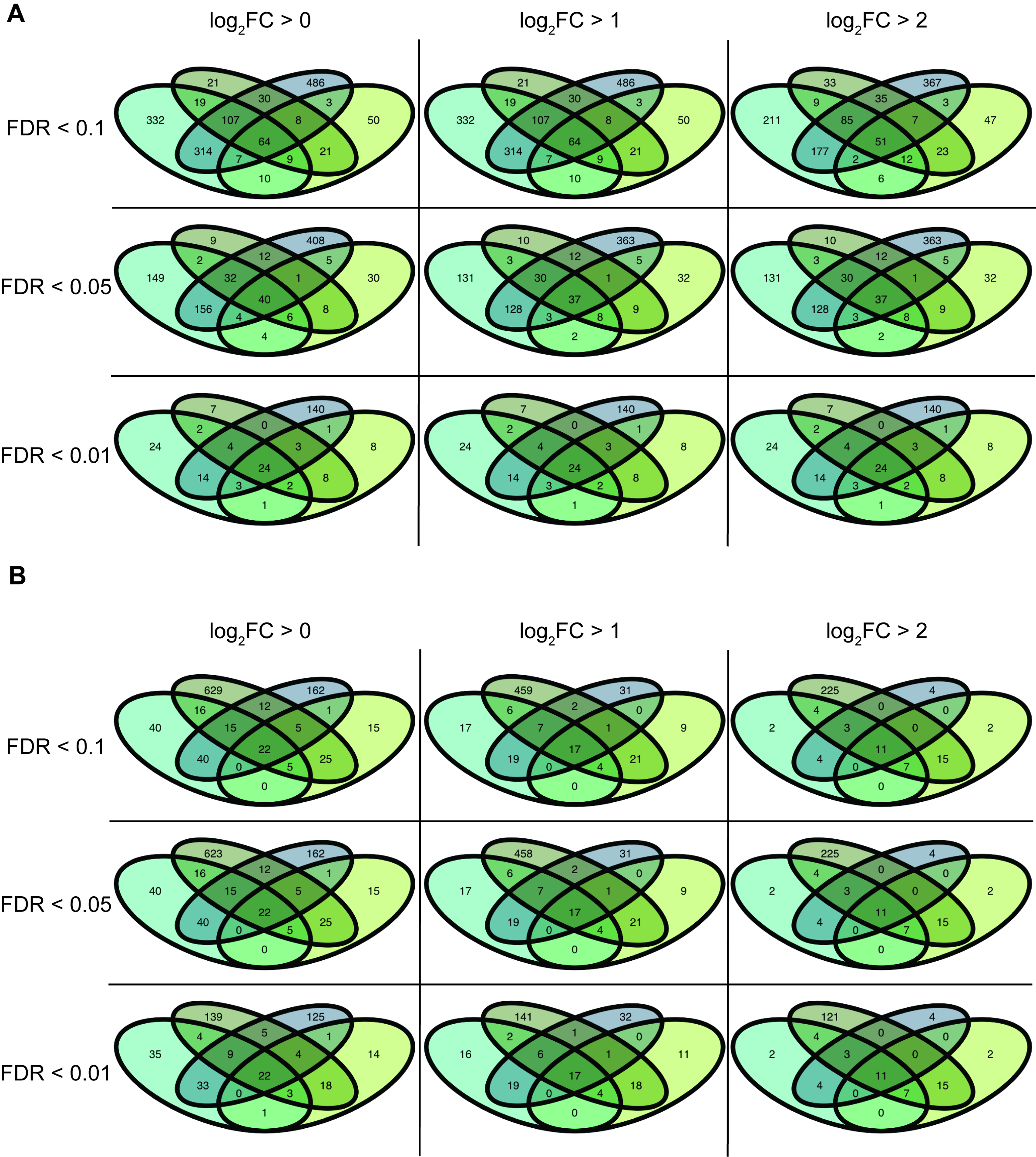

Supplement: S8 Fig — A). Overlap analysis between 24-hour RNA-seq in fat bodies when changing fold-change cut-offs along the y-axis (log2FC>0, log2FC>1, log2FC>2) and significance cutoffs along the x-axis (FDR<0.1, FDR<0.05, FDR<0.01). B). Same analysis as A, with hemocytes. (TIF) [file ppat.1011567.s008.tif]
