# Supplementary material for: Drosophila immune priming to Enterococcus faecalis relies on immune tolerance rather than resistance
Source: PLoS Pathog. 2023 Aug 11;19(8):e1011567. doi: 10.1371/journal.ppat.1011567 (PMC10446173; doi:10.1371/journal.ppat.1011567)
Supplement: Attachment — Submitted filename: PLoS Pathogens Review Response.pdf [file ppat.1011567.s015.pdf]

We would like to thank the reviewers for their feedback and suggestions on reviewing the manuscript. We have addressed each concern in Parts II and III in our point-by-point response below. Our responses are written in blue text.

Part I - Summary

Reviewer #1: In the manuscript entitled “Drosophila immune priming to enterococcus faecalis relies on immune tolerance rather than resistance” the authors develop a model for immune priming of Drosophila with Enterococcus faecalis and show that this results in tolerance of infection. They go on to conduct extensive RNAseq of the fat body and hemocytes at different time points in their infection models. They then subject IMD pathway, Toll pathway, and Eater mutants to their infection models and determine that IMD and Eater but not Toll play partial roles, suggesting that their priming phenotype is complex.

1). Priming is not a new finding, and it is not clear to me that the minor differences the authors point out between these observations and those of previous investigators reflect differences in microbe pathogenicity as opposed to differences in numbers of bacteria injected and time to second challenge.

2). While the extensive RNAseq data are likely to contain valuable information, they are not analyzed here with enough rigor or follow-up to yield testable hypotheses regarding the mechanism of priming.

3). Furthermore, the language in the manuscript is not precise and clear, the figures are not referred to accurately, and the statistics need to be more carefully explained or corrected.

Reviewer #2: In this manuscript, Cabrera and colleagues use Drosophila as a model to study immune priming in insect. They find that flies primed with a low dose of E faecalis survive better a challenge with a high dose of E faecalis. They further try to analyze the mechanisms underlying such priming effect. They find that priming is not correlated with a change in bacterial growth in the fly, suggesting it is a tolerance problem rather than a resistance problem. They further show that priming requires phagocytosis and IMD pathway. This paper is very well written and easy to follow. Their transcriptomics data are well documented and overall the idea that imd could be involved in an increase in tolerance during priming intriguing.

1). However, I do not feel that the few genes coming from transcriptomics that they single out at the end of the manuscript is the strongest conclusion to that story.

2). I feel that this paper is interesting and has potential to be published in PLOS Pathogens. However, I would want that results involving phagocytosis and the requirement of IMD are strengthened, and that tolerance is measured in these conditions before supporting the message of this paper fully.

Reviewer #3: The manuscript present an analysis of the priming of the innate immune system of Drosophila melanogaster with a low dose – high doses E. faecalis model. The first evidence for this is a low dose of E. faecalis (a Gram-positive) improves the survival of adult animals

against a second higher dose of bacteria. Further evidence indicates that this improvement is due to tolerance and not resistance, and priming requires the Imd pathway and phagocytic activity of hemocytes to work. On the other hand, The Toll pathway, itself responsible for the initial defense against E. faecalis, is not required for the priming. An RNAseq experiment reveals strong alteration in transcriptional profiles of fat body and hemocytes with priming and infection protocols, not surprisingly.

1). The authors' analysis of the RNAseq data claimed to reinforces the idea of tolerance being the driving mechanism of priming, but the underlying was not clear..

2). From the RNAseq data the authors identify genes that that stay upregulated throughout ("loiter"), which include several Toll pathway genes. The authors also identified some genes that turn off but get turned back on after the second infection ("recall") but do not elaborate on their roles or function in priming.

Part II – Major Issues: Key Experiments Required for Acceptance

Reviewer #1:

1) Figure 1B and 1C: One of the consistent observations from these experiments is that mock-primed bacteria die much faster than bacteria that are simply given one injection of high-dose E. faecalis. This is supported by the data in Figure 2 showing that the bacterial burden of mock primed flies on day 1 is two orders of magnitude higher than that of flies administered high dose Efae without mock priming. This suggests that mock-priming makes the fly more susceptible to infection, while priming with low dose bacteria makes the fly less susceptible. Is this the case? If so, how does this inform the mechanism of priming?

Thank you for pointing out this observation. It is true that mock-primed flies die ~1 day faster than flies given one high-dose injection of *E. faecalis*, however, there isn't actually a significant difference (Wilcoxon rank-sum, $p = 0.33$) between the bacterial load of high-dose and mock-primed animals one day post infection. (We realized this might have been confusing because panel 2A starts at day 0 – immediately after infection to confirm the difference in dose – while panel 2B starts at day 1. We have re-organized the panels to clarify this point). We feel hesitant to draw any strong conclusions based on the survival data alone, since the mock-primed flies are ~7 days older than the singly injected flies, so age may be a contributing factor.

2)

a) Here the authors graph expression of AMPs and Bomanins in the various conditions and comment that there is no difference between mock primed and Efae-primed. How was the synthesis of the data for all the time points and AMPs carried out? If the time points and AMPs were analyzed separately, would any significant differences be noted? Please give a rationale for lumping all the data together and describe how it was done.

The goal of this analysis was to assess whether the collective expression of either the AMPs or *Boms* correlated with survival. In our initial manuscript version, we decided to describe the overall strength of this response by combining the expression data of these gene sets using a geometric mean. We hypothesized that the collective expression level of, for example, the *Boms*, was more important that the expression of one particular family member.

To respond to this question, we considered doing pairwise comparisons of each AMP/Bom in the different conditions, but this quickly leads to a large number of comparisons that is hard to interpret. Therefore, we have reanalyzed the data and now show box and whisker plots with the individual expression levels of each gene and the median per experimental condition in both Figures 3E and 4B. The conclusion of this analysis, however, remains the same: every infection condition we considered shows an upregulation of these gene set families, but there is no significant difference in the distribution of expression levels of these genes between the Mock-primed and *Efae*-primed conditions. This suggests that the expression levels of these important immune effector genes are not driving the enhanced survival of the *Efae*-primed animals, which is consistent with the observation that the lack of difference of bacterial loads between *Efae*-primed and mock-primed animals.

b) Do these data come from the RNAseq data set? If so, it is not clear to me that a Welch's test is the correct one to apply. The significance test applied should be justified.

These data do come from the RNA-seq data set. In updating our analysis above, we have also shifted to presenting the data as a box-and-whisker plot and comparing between conditions using a Wilcoxon test. In this way we can display the full variability of the data along with the median. We choose a non-parametric test to be more conservative in our p-value calculations while also acknowledging that our data is not normally distributed.

c) The error in these measurements is quite large especially in Fig S2C. Is this a function of data aggregation? Does the unaggregated data yield a significant decrease in AMPs for Efae-primed flies at a particular time point, which would go along with the observed decrease in IMD in 1F? Even for the aggregated data, the trend is in that direction. Interestingly, based on 3F, one could also claim that there is no difference in IMD expression between uninfected and mock-primed flies, but the error bars are quite large. A follow-up qRT-PCR experiment might give more confidence in such statements.

The error in the previous measurements was in part due to the data aggregation. Consistent with the suggestion above, we have also modified Figure S2C to also show the spread of the data using a box plot instead of a geometric mean. The large spread is largely due to the fact that different Imd-responsive AMPs show quite a bit of variability in their fold-up regulation. This unaggregated method still does not show a significant decrease in Imd-dominant AMPs.

To the second point, the *imd* expression measurements in the day 7 control do show variability, however, using the differential expression pipeline employed in this work, a statistical comparison of the expression levels of *imd* in day 7 control vs. *Efae*-primed animals do show a significant difference (q-value = 0.046), which accounts for measurement uncertainty. More notably, the measured expression levels in the mock-primed and *Efae*-primed animals are not very variable, and also significantly different (q-value = 0.013), suggesting that this is a real difference in the *imd* expression response in *Efae*-primed vs. mock-primed animals.

Reviewer #2:

So far, I feel that the strength of the paper is to propose that phagocytosis and IMD pathways are involved in tolerance after priming. However, this currently is only supported by a mutant analysis in each case. Therefore I request some more data to support this model. Precisely, that means:

1- Get more than one mutant to demonstrate the requirement of phagocytosis. Additional hemocyte manipulation (genetic, ablation, using beads etc etc) is required.

Thank you for this suggestion. We have now included a bead blocking experiment to disrupt phagocytosis (Figure 1F). Consistent with the *eater* mutant, flies that have disrupted phagocytosis show worse survival than unperturbed flies. In fact, the flies with bead-blocked phagocytosis show among the worst survival assayed throughout the manuscript. The experiment is referred to in the text in Line 138:
"An orthogonal method of assessing the role of phagocytosis in priming – blocking phagocytosis with beads during the initial *E. faecalis* infection in OrR flies as was done previously (Pham, et al. 2007) – caused a complete loss of priming ability (Figure 1F)."

2- Get more than one mutant to demonstrate the IMD pathways is indeed required. I would recommend one additional way to alter IMD protein, and maybe to test additional mutants/KDs for other members of IMD pathway proteins.

We have included priming experiments for several Imd pathway mutants to further dissect the role of individual pathway components in producing a primed response. This includes single and double-injection experiments for the following mutant fly lines: *key*[1], *Tab2*[AOII3], and *Rel*[E20]. We found that like *imd* mutants, *Rel, key, and Tab2* mutants lost the ability to fully prime against *E. faecalis* infections. (By "fully prime", we mean that the primed animals survive as well as the PBS/PBS controls, as observed in the OrR wildtype flies.) The relative severity of the loss does depend on the mutant, with *Rel* mutants showing the weakest priming ability, followed by *key*, and then *Tab2* (which shows only a minor priming defect). Some of this difference may be due to *Tab2's* contribution to JNK signaling, but we lack the data to make any claims about this. These experiments strengthen the argument that intact Imd signaling is needed for full immune priming and is discussed in the text in Line 269:
"We further probed the role of the Imd pathway in immune priming and found mutants in three

additional pathway components, *kenny, Tab2,* and *Relish*, also show diminished immune priming (Supplementary Figure 4).”

3- It is unclear that when phagocytosis or IMD are lacking, tolerance is affected. The only measure of CFUs is done in the wild type, and it's only one proxy at one timepoint which may still be resistance. I suggest that in addition to what is currently provided, the authors measure the bacterial load upon death as published in Duneau at al, 2017. Alternatively, they could actually perform a regression analysis with multiple doses of pathogens and survival (the method implemented by David Schneider).

line 143: i think that BLUD data are needed to make this conclusion, or alternatively or more stringent measure of tolerance.

We have now included an experiment in which we have measured the bacterial load upon death (BLUD) in our OrR Mock-primed and *Efae*-primed flies (Figure 2C). We observed a significant increase in bacterial load upon death in our *Efae*-primed flies, supporting the conclusion from the initial bacterial load experiment that primed flies are tolerating an overall higher bacterial load than Mock-primed flies. We have included this analysis in the text Line 169:
“To further confirm that bacterial tolerance is driving the survival of *Efae*-primed flies, we also measured the bacterial load upon death (BLUD; Duneau, et al. 2017) for double-injected flies. The higher an animal’s BLUD, the higher its tolerance for a particular microbe. We found that *Efae*-primed flies harbored a significantly higher bacterial burden at the time of death (Figure 2C).”

4- They should redo their measure of tolerance in their mutants of phagocytosis and IMD pathway. This is unclear to me that these mutants actually lead to a change in tolerance, and this is inferred based on WT, but not demonstrated currently.

We have now tracked bacterial load after re-injection in both our *imd* and *eater* mutants. We would expect that if resistance was impaired in these mutants, the bacterial load would be higher in the mutants than the wild type, OregonR, animals.

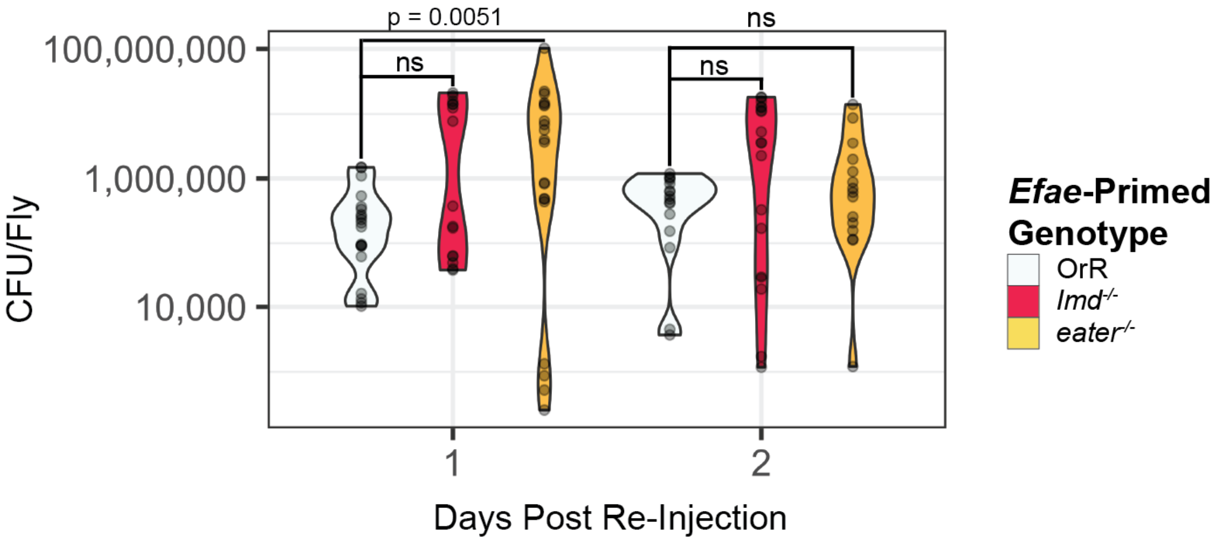

This analysis proved to be somewhat challenging, as there was an even stronger survivor bias in these mutants as compared to our OrR control. We therefore have limited our analysis to the first 48 hours post re-injection. At day 1, only the *eater* mutant shows an increased, though variable, bacterial load as compared to OrR, while at day 2, there is no significant difference, suggesting that resistance is not strongly impaired in these mutants. However, to ensure that we are not overstepping the bounds of our data, we have also softened the language about tolerance in the mutants on lines 167-175.

Reviewer #3:

1. Figure 1C, 1E, 3H, 5E: The key comparison and conclusions in this study rely on qualitative examination of survival curves and median survival time (without statistical testing) to support the authors' arguments. However, the actual statistical analysis, as presented, do not support these conclusions. The P-values from the log-rank test of survival curves indicate that all mutants have similar priming activity (usually with P-values marked with ***), which contradicts the main claims the authors make. The authors may be correct in their intuitive analysis of the data, but the statistics are inadequate for their arguments. For example, it does not appear that the log-rank analysis was corrected for multiple comparison which might result in an analysis consistent with the authors intuitive conclusions; the mean survival time should be statistically analyzed, and/or perhaps a quantitative analysis of the hazard ratio might be informative. Regardless, the statistical testing needs to support the main conclusions.

Thank you for this suggestion. We have shifted our analysis to use hazard ratios to compare survival curves in a more rigorous fashion. We use them in two contexts. First, we compute the hazard ratios (HR) of infected animals compared to a single or double PBS control to measure how the pathogen affects survivors as compared to the negative PBS controls. We have also calculated HRs comparing survival in *Efae*-primed flies to Mock-primed flies. We define priming as increased survival when comparing *Efae*-primed to Mock-primed flies (HR < 1), and "full" priming as when *Efae*-primed animals survive as well as the PBS/PBS control (HR ~ 1). In this

way, we can assess not only whether there is a statistically significant difference in survival between the two, but also the intensity of that difference across time. We also report the median survival time to comment on the temporal survival dynamics. We have updated all our figures accordingly and summarize our survival data in Supplementary Table 1.

Using this updated strategy, we still find that OrR flies are able to prime against subsequent *E. faecalis* infection (Efae-primed vs Mock-primed, HR = 0.28 [95%CI 0.20-0.41]), and that training with heat-killed *E. faecalis* produces a suboptimal priming phenotype (HR = 0.52 [95% CI 0.37-0.74], HR is higher than training with live *E. faecalis*). Comparing *Efae*-primed survival to Mock-primed survival, we find that both *imd* mutants (HR = 0.39 [95% CI 0.27 - 0.58]) and *Myd88* mutants (HR = 0.32 [95% CI 0.20 - 0.53]) are still able to prime. However, despite being in the same genetic background, *imd* mutants are not able to recapitulate OrR's ability to match *Efae*-primed survival to PBS/PBS survival (*Efae*-Primed vs. PBS/PBS; OrR HR = 0.75 [95% CI 0.53 - 1.1], Imd HR = 4.1 [95% CI 2.7 - 6.3]. This more-nuanced interpretation supports our conclusion that loss of Imd reduces the "full" priming capacity against *E. faecalis*.

2. The wild type background of the survival experiments are not properly controlled. Additionally, one case (1C & 1E) it seems that the PBS/PBS groups have vastly different response. This undermines the ability of the authors to make claims about the contribution of each pathway and process toward priming. To be explicit, none of the mutant strains analyzed are in the Oregon R background; a matched wildtype needs to be included in all analyses.

We agree that the genetic background is an important consideration. In this manuscript, we have used several mutant lines including *eater*, *imd*[10191], and *Myd88*. The *imd*[10191] strain was backcrossed to the OregonR background, and therefore doesn't suffer from the same caveats at the *eater* and *Myd88* mutant lines. Although the ideal approach would be to backcross these mutants into the OregonR background, this is a lengthy process, and unfortunately we were unable to obtain a reference strain for the *Myd88* mutant. Therefore, we have addressed this concern in several ways:

1. For all survival analysis, we now calculate the hazard ratios, as suggested above. Although this is not a perfect control for genetic background, it does allow us to assess the relative hazards of infections against an appropriate PBS or PBS/PBS injection control or the Mock-primed condition.
2. For the *eater* mutant, we have further investigated the role of phagocytosis using a bead feeding experiment as summarized in our response to reviewer 2 above (results in Figure 1F in the text). Using this method, we were able to disrupt phagocytosis in our OrR control background and show these animals are unable to be primed against a subsequent infection. The results of this experiment supported our original hypothesis that phagocytosis during the initial *E. faecalis* infection is necessary to induce priming against a second *E. faecalis* infection without the potential caveat of genetic background.
3. For the *Myd88* mutant, we have further investigated the role of the Toll pathway in priming by assaying survival in a *spätzle* mutant. We found that although *spz* mutants and *Myd88* mutants respond similarly to a single *E. faecalis* infection (Figure 5D & 5F), *spz* mutants lose the ability to prime entirely (Figure 5G). This was somewhat surprising

as it indicates that components of the Toll pathway are not completely dispensable for a primed immune response. The results of this experiment are expanded upon in the text and discussion.

Line 351 in Results: “We additionally parsed the effects of eliminating extracellular Toll signaling versus intracellular signaling by assaying *spz* mutants. Similar to *Myd88* mutants, we found that ablating *spz* maintained the dose-dependent response to *E. faecalis* single infections (Figure 5F; Efae Low Dose vs PBS HR = 2.9 [1.9-4.5], Efae Hi Dose vs PBS HR = 6.1 [4.1-9.1]). However, the *spz* mutants lacked the ability to prime against *E. faecalis* (Figure 5G; *Efae*-Primed vs Mock-Primed HR = 1.2 [0.83-1.7]). This indicates that immune priming against the Gram-positive *E. faecalis* does not strictly require Myd88-mediated Toll signaling, but does require extracellular Spz activity.”

Line 469 in Discussion: “The precise mechanism driving this difference between the Toll mutants remains unclear – the response of the *Myd88* and *spz* mutants to a single injection, whether of PBS, or a high or low dose of *E. faecalis* are remarkably similar. Further, their response to the PBS/PBS or Mock-priming dual injections are also virtually indistinguishable. This implies some extracellular activity involved in upstream Toll signaling is necessary to mount a primed immune response, but that Myd88 intracellular activity is not and suggests further probing into the underlying mechanism is warranted.”

3. The visual and labels used in figures 3 and 4 are clear on the conditions used in the RNAseq experiment. However, this text is completely muddled, when the authors attempt to describe the analysis and results, and this reviewer was unable to follow the narrative.

We apologize for the confusion within the text. We have edited the text throughout to try and clarify the conditions in each instance.

4. The authors also claim that hemocytes act as signal relayers for the priming action, but this claim is “synthesized” from two unrelated experiments and the authors do not attempt to explain how the phagocytosis activity of hemocytes can work as a signal relaying mechanism or how this might play into the improved tolerance which is suggested as key mechanism for priming. In general, the manuscript lacks a thorough mechanistic probing of the phenomenon presented.

We agree that the paragraph synthesizing the hemocyte observations is more suitable for the discussion than the results, and have edited the manuscript accordingly. We have now moved and edited the paragraph to soften the claims on Line 386:

“Overall, we have seen evidence for tolerance, phagocytosis, and transcriptional reprogramming as a drivers of priming against *E. faecalis* infection. Flies primed against *E. faecalis* re-infection did not actively clear bacteria more efficiently than Mock-primed controls (Figure 2B), and did harbor a higher bacterial load upon death (Figure 2C), both hallmarks of infection tolerance. We also found that phagocytosis was needed in order to fully prime, as supported by the decrease in priming ability in both our *eater*-deficient flies (Figure 1E) and bead-blocking experiments (Figure 1F). Given that primed flies seem to survive infection by tolerating, rather than clearing bacteria, this suggests a role for phagocytes in priming other than their canonical responsibility

of eliminating pathogens. One possibility is that phagocytes are working to sense an infection and relay that signal to other tissues through functional reprogramming (Nehme, et al. 2011; Gold & Brückner 2014). This is supported by the large transcriptional shift in metabolic pathways seen in hemocytes, and specifically, the up-regulation of lysozyme-related pathways, including the “MHC Class II Antigen Presentation” and “Neutrophil Degranulation gene sets (Figure 4C). Explicit proof of phagocyte reprogramming as a potential mechanism of priming merits further investigation. Transcriptionally, there are three primary mechanisms suggested that may underlie immune priming – (1) primed animals may drive a qualitatively different expression program than mock primed flies, differentially regulated distinct genes, (2) primed flies may continually express key immune genes between a priming and subsequent infection, or (3) primed flies may re-active an immune response more quickly than unprimed flies. Our transcriptional data shows that most priming differences in both fat bodies and hemocytes can be attributed to gene expression that is unique to priming (Figure 3B & 4A). We saw continuous expression of a small number of Toll effectors in fat bodies (Figure 5B), and very little evidence of potentiated gene expression (Figure 6B).”

5. The authors suggest that improved tolerance is responsible priming, but aside from bringing up metabolism in RNAseq analysis while discounting the possible roles of loitering or recall genes, the authors do not further attempt to explain or suggest a mechanism for bacteria tolerance

We agree this is an important point to probe further. As described above in response to reviewer 2, we have more carefully probed the role of tolerance by measuring bacterial loads in several new conditions and by carrying out an experiment to measure bacterial load upon death (BLUD), which is the current gold standard for measuring immune tolerance. We found that *Efae*-primed flies harbored a significantly higher bacterial load upon death compared to Mock-primed flies. This is consistent with our initial assessment that *Efae*-primed flies are more actively tolerating the infection. As for the mechanism of what specifically is driving the improved tolerance, we agree this would be of great interest. We have added a revised paragraph in the discussion to more clearly highlight what we have learned in this work (see response to the point just above.) We also suggest that the lists of priming-specific upregulated genes would be a useful starting point for further dissection of the mechanism, and are working to use other analysis tools to prioritize the list, but given the number of potential targets, feel that testing all these gene candidates is beyond the scope of this manuscript.

6. The GSEA visual and methodology is quite novel, and yet it is too complex and unclear. The authors should attempt to reduce the complexity and explain the meaning behind the analysis and the visualization, as well as how each claim is drawn.

We appreciate the reviewer’s opinion on the text and interpretability of the figure. We have now expanded the figure, figure legend, and main text to include a more detailed description of the analysis used.

Line 206 in Results: “GSEA is an approach that looks for the coordinated up- or down-regulation of a set of genes involved in a common pathway or function. Since it uses all the transcriptome data, as opposed to differentially expressed genes identified by a fixed threshold, it can reveal

differentially expressed pathways between samples that GO analysis may not detect."
Line 309 in Results: "Given the diverse functions of hemocytes in immune response, we decided to use GSEA to again systematically delineate priming-enriched pathways (Figure 4C, full GSEA analysis in Supplementary Table 65). Figure 4C shows individual gene sets enriched in either *Efae*-primed or Mock-primed hemocytes as nodes whose size represents the proportion of genes within a set that were found to be enriched. Edges connect nodes that share overlapping genes between gene sets, and their thickness represents how many genes are shared. This analysis of hemocyte transcription in *Efae*-primed samples versus Mock-primed samples indicated a wider picture of metabolic reprogramming (Clusters 2, 6, 8, 10, 11, and 13) and altered protein production (Clusters 4, 5, 6, and 7) in the primed samples. There was also enrichment for genes involved in antigen-presenting and neutrophil degranulation functions in mammalian orthologs, which contained several lysosomal and metabolic genes associated with bacterial immune response, such as the GILT family of genes."

Part III – Minor Issues: Editorial and Data Presentation Modifications

Reviewer #1:

1) Introduction, line 44: I am not sure what "qualitatively different" means here.

In this instance 'qualitatively different' refers to an immune response that, for example, differs in the identity of what effectors are produced rather than varying in the intensity of response. We have modified the sentence on line 43 to now read:
"The first is that there is a qualitatively different response, e.g. a difference in the identity of the effectors produced or cellular processes, between primed versus non-primed insects, leading to a more effective response."

2) Figure 1 C and D: The Efae-primed survival data for Figs C and D look very similar. If they are from the same experiment, these graphs should be combined, or the authors should note in the Figure Caption that the same data were used in both figures.

The text in the figure legend has been modified to indicate that the data for OrR *Efae*-Primed survival is the same between Panels C & D. It now reads:
"D). Survival of OrR flies primed with heat-killed E.faecalis (EKill-Primed: n = 55) versus flies primed with live E.faecalis: p = 0.00068; data for Efae-Primed same as Panel C."

3) Line 105, Fig 1E: The authors conclude that the differential response to live and dead bacteria suggests a mechanism other than bacterial sensing. Another possibility is that bacterial sensing is required throughout the seven-day priming interval, and the products of the dead bacteria are cleared rapidly and no longer activate bacterial sensors.

Thank you for pointing out this possibility. We have now modified the sentences starting on line 116 as follows:

“To see what bacterial signals are required for priming, we attempted to prime flies with heat-killed E. faecalis, which retains its signaling-responsive components but lacks any additional virulence factors (Itoh, et al. 2012; Adams, et al. 2010). This experiment resulted in a more moderate increase in survival rate compared to live bacteria priming (Figure 1D, HK-Efae-Primed vs. Mock-Primed HR = 0.52 [0.37-0.74]). As can be seen by comparing the HK-Efae-Primed survival curve to the PBS/PBS survival curve, these animals do not achieve “full” priming as is the case with live bacteria. This implies some level of priming is conferred simply through bacterial sensing, but that the effect is not as robust as when the fly is exposed to the live microbe. This may either be because the live microbe produces other virulence factors or damage that is needed for priming or because the heat-killed microbe’s products are cleared too quickly to create an equally strong priming response.”

4) Line 109, Figure 1E: These data are referred to again at the end of the results section. The authors should consider moving all discussion of these data to the section at the end of results.
In response to reviewer #3’s comment #4 above, we decided to remove this paragraph from the results and put them in the discussion section instead. Therefore, Figure 1E is now only discussed in the results section in the beginning.

5) Line 140 and Fig 2B: A time course for bacterial burden is shown but only one p-value is given in the text. Which time point does this p value refer to? Also, it is most informative to indicate p values on the graph for all comparisons in all figures.
The Kruskal-Wallis test used in Figures 2A & 2B tests for overall difference in bacterial load over time, rather than on a day-by-day basis. It is non-parametric version of the one-way ANOVA – it looks for differences between the two experimental conditions (e.g. Low Dose vs. High Dose) on each of the time points and reports a p-value indicative of whether there is a difference on any of the days. We have modified the text to indicate this, and p-values have been added to the graphs in Figure 2.

6) Line 167, Fig 1B: The fat body GO analysis terms highlighted refer to general processes such as immune response, response to stress, and cell surface receptor signaling. Without follow-up experiments, these categories are not particularly informative.

We agree that GO analysis is a bit high level and vague. Since it was not central to our narrative, we chose to move the graph to the supplementary material (SuppFigure 2) and de-emphasize it in the text. Revised now in Line 196: “Only a small fraction of these genes have been previously annotated with immune functions (19 Efae-Primed genes, ~13%; 15 Mock-Primed genes, ~4%) (Ramirez-Corona, et al. 2021; Troha, et al. 2018), although gene ontology (GO) analysis indicated immune response as one of the highest enriched terms (Supplementary Figure 2B, top).

7) Figure 3: It is difficult to remember the color-coding scheme for the different experimental conditions studied. I suggest including a key in every figure in which the color coding is used.

We have reviewed all figures to ensure they have a key that describes each color used. It has been indicated in the figure legend that all colors match the conditions in Panel A.

8) Figure 3E and F and S2C:

We were unsure of the comment here, but these figures have been revised.

9) Abstract, line 15 and throughout: The phrase “loitering genes” is vague. The genes are present in all cases. They are persistently differentially regulated. In line 49, the authors state the effectors loiter. Has the concentration of effectors been measured? I suggest the authors use terminology that describes the phenomenon in place of the term “loiter.”

To more accurately reflect the analysis done to classify these genes, we have changed to using the phrase “continually express” rather than “loiter”.

10) Introduction, line 44: I am not sure what “qualitatively different” means here. Did the authors not measure the response?
In this instance ‘qualitatively different’ refers to an immune response that, for example, differs in the identity of what effectors are produced rather than varying in the intensity of response. We have modified the sentence on line 43 to now read:
“The first is that there is a qualitatively different response, e.g. a difference in the identity of the effectors produced or cellular processes, between primed versus non-primed insects, leading to a more effective response.”

11) Introduction, line 50: I would eliminate “often” here unless this has been shown to be a frequent state of flies.

Rephrased to ‘can harbor’ in the text. Line 51

12) Figures 2A and 1C are referred to out of order.

We realize this is the case, but wanted to keep the bacterial load data in a single figure, for ease of comparison later on in the results. We hope this is acceptable.

13) Line 271: Figure 3C should be Figure S3C. Furthermore, I do not see data for an Eater mutant in Figure 2B.
The text is referring to Figure 4C. It has been corrected in the text.

14) Line 280: Authors refer to Figure 1F, which does not exist. This should be Figure 1E.
Corrected to ‘Figure 1E’ in the manuscript.

15) Line 338: Myd88 mutants have no effect. This is not “an unexpected effect.” This should be rephrased.
Rephrased in the text in Line 380. It now reads:
“When testing priming ability in *imd*, *Myd88*, and *spz* mutants, we found that these mutants have unexpected survival phenotypes in the double injection conditions – *imd* mutants prime less effectively than wild type flies, *Myd88* mutants show no apparent loss of priming ability, and *spz* mutants completely lose the ability to prime.”

Reviewer #2:

1). line 99: fig1C, Ef primed - PBS injected control is missing; what about other 'wt' strains? is this effect OrR specific or general? PBS/PBS effect seems to be dramatic for OrR (but not for Eater-/)

We have discussed further implications of the genetic background and how we analyze this in response to Reviewer #3's comment #2 in the "Major Issues" section.

We have also refined our survival analysis by using hazard ratios (HR). We define the ability to prime as the ability for Efae-Primed flies to survive better than Mock-Primed flies (i.e. *Efae*-Primed vs Mock-Primed HR < 1). We also define "full" priming as the ability to match *Efae*-Primed survival rate to PBS/PBS (i.e. *Efae*-Primed vs PBS/PBS HR = 1). This approach has been elaborated on in the text in Line 104:

"We define priming as an increase in survival in *Efae*-primed flies compared to Mock-primed flies. Quantitatively, we assessed priming by comparing *Efae*-primed to Mock-primed survival using the HR; priming is indicated by a HR that is significantly less than 1"

Line 110: "We can again use the HR to define this "full" priming – when the *Efae*-primed flies survive as well as the double-PBS control, this results in a HR that is not different from 1."

2). line 113: seems to be very similar to immune priming effect seen in OrR (fig1C)

OrR 50% lethality (Ef primed - Mock primed): 4d - 1d

Eater 50% lethality (Ef primed - Mock primed): 3d - 1d

OrR - Eater: is the difference significant?

As mentioned above, to more carefully analyze our survival curve data, we have hazard ratios for the different infection conditions/genotypes, accounting for the appropriate PBS or PBS/PBS control. The answers to the questions above are now explained on Line 133:

"By comparing the *Efae*-primed to Mock-primed flies, we can observe a modest amount of immune priming, with a median survival time of 3 days and 1 day, respectively (Figure 1E). However, the *Efae*-primed flies have a shorter median survival time than the PBS/PBS controls. This trend is mirrored by the HR comparing *eater Efae*-primed flies to the PBS/PBS control, which is greater than 1 (HR = 3.8 [2.3-6.4], Supplementary Table 1), indicating that the mutants are unable to achieve full priming. An orthogonal method of assessing the role of phagocytosis in priming – blocking phagocytosis with beads during the initial *E. faecalis* infection in OrR flies as was done previously (Pham, et al. 2007) – caused a complete loss of priming ability (Figure 1F). Together, this indicates that phagocytosis is needed to fully prime."

3). line 116: "indicating that phagocytosis is needed to allow Efae-primed flies to survive": i am not sure this is valid. Could the authors elaborate?
We have now interpreted this data a bit more precisely, as written in the quoted text in response to the point #2 above.

4). line 143: i think that BLUD data are needed to make this conclusion, or alternatively or more stringent measure of tolerance.

To strengthen our understanding of tolerance in immune priming, we have measured bacterial load upon death (BLUD) in double-injected OrR flies. The results from this experiment are visualized in Figure 2C and indicate that tolerance is underlying the enhanced survival of the primed flies.

5). line 151: how different are these samples if we compare global gene expression data?

We compared our transcriptional response to the whole-organism approach taken in Troha, et al. 2018 in Supplementary Figure 2A. In that study, the authors identified a core set of genes that were up-regulated across several types of bacterial infections (including *E. faecalis* infection). We then surveyed if we observed up-regulation of that core gene list across all of our fat body samples and found that a little under half of that core gene list was up-regulated across our samples, with a subset being up-regulated in some conditions, and some genes not being up-regulated in our samples at all. To our knowledge, there's not an equivalent gene expression data set for priming with *E. faecalis*.

6). line 159: it would be useful to see gene names / clusters
Previously annotated immune genes are indicated in Supplementary Table 3 (for fat body samples) and Supplementary Table 5 (for hemocytes). An additional sheet (AllCond_ImmunoCore) has been added in each table to include core immune genes from Troha, et al. 2018 that are up-regulated across all conditions.

7). line 228: i see difference between OrR and imd mutant: why do they conclude that imd makes no impact?
We have been able to refine this result by including hazard ratios. The full details are in the text, but briefly, in both the OrR and *imd* mutant, animals infected with the low dose of *E. fae* compared to the PBS control have hazard ratios not significantly different from 1, indicating that the low dose doesn't cause death at much of a greater rate than a sterile wound, while the high dose is much more lethal. This can be compared to the case of the *Myd88* mutant, where the low dose animals have a HR of 4.2 – indicating that they die at a much faster rate than the PBS control

8). line 230: would it make sense to compare the survival after high dose Ef in G and high dose survival in H?
We are hesitant to draw strong conclusions from comparing between flies singly injected with *Efae* High Dose and *Efae*-Primed flies due to potential age-related effects.

9). line 233: based on high dose Ef injection on 3G: it does. What do the authors think?
The survival in *imd* mutants against both a low-dose and a high-dose *E. faecalis* infection is comparable to an OrR control line. We have rephrased the text as follows in Line 261:
"As has been previously shown, the *imd* mutant showed a dose dependent response to *E. faecalis* infection with levels of lethality similar to a non-immunocompromised OrR control

(Figure 3F & Supplementary Table 1; OrR HRs [Low Dose = 1.4 (0.97-2.1) , High Dose = 5.7 (3.9-8.3)] & *imdIMD* HRs [Low Dose = 1.3 (1.0-1.8), High Dose = 3.3 (2.4-4.6)]).

10). line 235: "This suggests that there are distinct differences in use of signaling pathways between animals with one versus two infections." i am not sure about this conclusion. Could the authors develop their thought or bring more precision?

We decided to remove this sentence and save further analysis for the discussion section.

11). line 245: how different are these samples if we compare global gene expression data?

Unfortunately, there is no equivalent global gene expression data that has the same experimental conditions to which we can compare.

12). fig3B and 4E: AMP + Bom profile show dramatic difference: the authors should explain or at least mention and comment it in the text

We have decided in response to a comment above (reviewer #1, major issue #2a) to alter the way we approached the analysis in these figures and have updated the text accordingly.

13). line 279: there is only a minor difference, i am not sure it is significantly different (eater-/- data)

In response to reviewer #3, major issue #4, we have decided to remove this paragraph.

14). Line 280: "indicates that…": based on data, i am not sure I see that.
In response to reviewer #3, major issue #4, we have decided to remove this paragraph.

15). lines 282-283: it is too simplified, there are several differences between samples: d1 and d8 FB are different ; d1 and d8 Hemocyte are different etc. Could that be further described/analyzed?
In response to reviewer #3, major issue #4, we have decided to remove this paragraph.

16). lines 285-287: i am not sure this is true based on their data
In response to reviewer #3, major issue #4, we have decided to remove this paragraph.

17). line 308: figS5A: are OrR data the same as shown in FigS2E? If yes, please redo.
The Oregon-R data is the same for both of those figures. We have now clarified this in the figure legend. Since each survival curve is based on at least 60 animals that were infected in at least 2 separate experiments, we feel confident in the data shown.

18). line 317: I somehow feel this is a weak ending. I see the value of that group of genes, but I feel somehow biological conclusions on the functional genetics that has a phenotype is more impressive.
We agree that it would have been exciting to explore genes that appear to be more strongly/quickly up-regulated in response to re-infection, suggesting perhaps an epigenetic change to their promoters or enhancers. However, given the very small number of genes revealed in this analysis, we did not find a great candidate for followup. We considered

removing this section, but since it’s often proposed as a model for how priming might occur, we felt there was value in keeping it in. We also considered moving it to earlier in the manuscript, but thought it might take away from other more exciting sections. Therefore, we decided to leave it as is, but are open to other suggestions.

Reviewer #3:

1). Line 280: Figure 1F does not exist
Corrected to ‘Figure 1E’ in the manuscript.
