# Supplementary material for: Drosophila immune priming to Enterococcus faecalis relies on immune tolerance rather than resistance
Source: PLoS Pathog. 2023 Aug 11;19(8):e1011567. doi: 10.1371/journal.ppat.1011567 (PMC10446173; doi:10.1371/journal.ppat.1011567)
Supplement: Attachment — Submitted filename: Response Letter #2.pdf [file ppat.1011567.s016.pdf]

We would like to thank the reviewers for their feedback and suggestions on reviewing the manuscript. We have addressed each concern in our point-by-point response below. Our responses are written in blue text.

Part I - Summary

Please use this section to discuss strengths/weaknesses of study, novelty/significance, general execution and scholarship.

Reviewer #1:

In the revised manuscript entitled “Drosophila immune priming to enterococcus faecalis relies on immune tolerance rather than resistance” the authors have addressed some of the concerns brought up by the reviewers. It is not clear that the new statistical tests applied are more appropriate than the ones applied previously or that multiple comparisons have been taken into consideration. The addition of beads to the phagocytosis data provides additional confidence, but the spz data raise questions. As such, the data remain a collection of observations about the role of phagocytosis, IMD pathway, and spz in immune priming that do not clarify the process under study.

We appreciate this reviewer’s feedback and are glad to hear that they feel that we have addressed some of the reviewers’ concerns. With respect to the comment “It is not clear that the new statistical tests applied are more appropriate than the ones applied previously or that multiple comparisons have been taken into consideration”, we have chosen to use the hazard ratio analysis because it allows us to quantify whether two survival curves differ and also provides a quantitative summary of the degree of difference, as suggested by Reviewer #3 in the previous round of reviews. The associated p-values have been corrected for multiple comparisons, as detailed below. Please also see below for a response to your comment about our *spz* data.

Reviewer #2:

I feel that the authors have successfully tackled my major comments

Reviewer #3:

Cabrera et al. presents a characterization of the priming of Drosophila melanogaster innate immune system by Gram-positive E. faecalis. Adult animals subjected to a low dose of E. faecalis have improved survival against a second higher dose of the bacteria. The authors present evidence showing increased bacteria tolerance and phagocytic activity are key mechanisms of priming. The RNAseq analysis reveals interesting transcriptional changes involving metabolic shift associated with priming.

Part II – Major Issues: Key Experiments Required for Acceptance

Please use this section to detail the key new experiments or modifications of existing experiments that should be absolutely required to validate study conclusions.

Generally, there should be no more than 3 such required experiments or major modifications for a "Major Revision" recommendation. If more than 3 experiments are necessary to validate the study conclusions, then you are encouraged to recommend "Reject".

Reviewer #1:

1) For all bacterial load experiments, the authors should consider applying their statistical test after log transformation of the data. This is a commonly used approach to skewed data and, in particular, bacterial load measurements that reduces the influence of outliers.

Thank you for this suggestion. We realized it wasn't clear that the rank-sum tests used in Figure 2 were calculated using the log-transformed data, and we added the following sentence to the methods to help clarify this:

Line 557: "Comparisons between bacterial loads were done using rank-sum tests on log-transformed data."

2) In Figure 1C and D, the investigators have moved to a hazard ratio instead of log rank analysis without providing justification other than that this is "more rigorous." Has the author has modified the test for multiple comparisons as recommended by reviewer 3? In fact, although the survival traces are separated in Figures 1 C and D, since the authors acknowledge that these two panels utilize the same control traces (PBS/PBS and mock primed), a statistical test must take all four conditions (two controls, two tests) into account in the multiple comparison analysis regardless of how the data are presented.

As mentioned above, we have chosen to use the hazard ratio analysis because it allows us to quantify whether two survival curves differ and also provides a quantitative summary of the degree of difference, as suggested by Reviewer #3 in the previous round of reviews. We have described its use here:

Line 96: "Survival was scored as the hazard ratio (HR) of the bacterial-infected flies against a PBS-injected control; a HR > 1 indicates worse survival of the experimental sample compared to the control. The HR also gives a quantitative summary of the survival curve – higher the HR, the more quickly the animals died."

We have used a Benjamini-Hochberg correction to ensure the p-values are appropriately adjusted for multiple test comparisons. These corrected values are displayed in Supplementary

Table 1, Figure 1, and Figure 3 and mentioned in the Methods section.

3) Fig 3D and E and 4B: It is not clear to me that a pairwise statistical test can be applied to data extracted from an RNAseq experiment that involved multiple comparisons. While applying the appropriate test would not change the significance result, it is important to use statistics correctly.

Please accept our apologies for the confusion: the data used for this analysis was not resultant of the EdgeR analysis (which requires multiple tests as the reviewer suggests), but rather is the expression levels of a selected group of genes measured using transcripts per million (TPM) values. To compare these TPM values across conditions, we chose to use a Wilcoxon test, as opposed to a t-test, because sample size was too small to assume central limit theorem, and there's no indication that the genes will be normally distributed. The p-values were corrected for multiple testing using Bonferroni correction, which is now mentioned in the figure legend and methods section.

4) The new data suggesting that Spz is required for priming but not Myd88 is confusing and requires more follow-up to be certain this is not an artifact of the mutant used, etc.

We were also puzzled by the discrepancy between the Spz and Myd88 mutants. We agree that to definitely tease apart what is happening here, the genetic backgrounds need to be more carefully controlled, ideally by back-crossing both mutations into the Oregon R background. Given the timeline required of this revision and the fact that this data does not affect the main conclusions of our study, we felt it most appropriate, and potentially helpful to other researchers working with these strains, to move these survival curves into the Supplementary Material and include information about the caveat in their interpretation. We thank the reviewer for pointing this out.

Reviewer #2:

No experiment is required at this stage

Reviewer #3:

1. The authors' previous analysis and conclusion implied Toll not being required for priming despite being essential for surviving the first infection. On the other hand, the Imd pathway was argued to be required for priming based. However, even with newly provided data the revised analysis does not seem to back up this claim, and frankly lacks statistical rigor.

Line 280-282: The authors claim that kenny, Tab2 and Relish mutants, similar to imd, have diminished immune priming. Upon closer inspection of the Efae Prime/Efae Mock in these data,

only Rel[E20] seems to have a notably different priming Hazard Ration (HR=0.606) compared to OrR (HR=0.2855), albeit with a major caveat that the genetic backgrounds are not controlled. However, the Efae Primed/Efae Mock HR of imd (0.3942), kenny (0.2777) and Tab2 (0.3951) are very similar to Myd88 (0.3241), which the authors consider to have normal priming, and to OrR (wildtype). Just a curiously examination of these survival curvies, Figure S4, suggests priming is intact in most of the imd pathway mutants. Moreover, the comparison of Hazard Rations between these different mutants lacks rigorous statistical testing.
It seems that the authors still retained the conclusions drawn from the comparison of median survival time but have taken most of the sentences that mentioned this parameter out of the reasoning due to the lack of statistics. The authors should consider include median of survival time back (with statistics), or exclude it completely and draw new conclusion?

Thank you for the careful commentary on this analysis. In the case of the *imd* mutant (which is in the OreR background), we find that the mutant can still prime (HR *Efae* primed vs. mock = 0.39), it does not prime as well as OreR (HR *Efae* primed vs PBS/PBS = 4.14 for *imd* mutant, as compared to 0.75 for the OreR control). We describe this in the text:

Line 275: “However, when subjecting the flies to dual injections, we observed a significant, though not total, loss of priming ability in these *imd*-mutant flies (Figure 3G). *Efae*-primed flies still survive a second injection more effectively than Mock-primed flies (*Efae*-Primed vs. Mock-Primed HR = 0.39 [0.27-0.58], p = 2.5e-6), but less successfully than control flies twice injected with sterile PBS (*Efae*-Primed vs. PBS/PBS HR = 4.14 [2.7-6.3], p = 5.3e-12). This is in contrast to wildtype OregonR flies, which survive a secondary *Efae* infection as well as a double wounding (OreR *Efae*-Primed vs. PBS/PBS HR =0.75.)”

Although there is notably the caveat of genetic background, the *kenny*, *Tab2* and *Rel* mutants all show HR’s of E fae primed vs. PBS/PBS controls that are significantly greater than 1 (HRs = 2.3, 2.0, 3.7, respectively, with p-values <0.05). We have edited the sentence in the manuscript to clarify this point:

Line 281 “We further probed the role of the Imd pathway in immune priming and found mutants in three additional pathway components, *kenny, Tab2,* and *Relish*, also show diminished immune priming, as indicated by HR > 1 when comparing the *Efae*-primed flies to the PBS/PBS control (Supplementary Figure 4).“

2. The lack of thorough consideration of and control for genetic background remains an issue. It is not at all clearer why OrR is an appropriate control for any of the mutants examined.

We use mutants in three places in the manuscript – to test the role of phagocytosis, to test the role of IMD signaling, and to test the role of Toll signaling. In the case of phagocytosis, the concordance between the *eater* mutant and bead blocking experiment, which was done in the OregonR genotype, gives us confidence in the role of phagocytosis in priming experiments. However, given the relative strength of these two pieces of evidence, we have revised the manuscript to put the bead blocking experiments first in the text and figure and include a brief phrase acknowledging the genetic background of the *eater* mutant.

For the case of IMD signaling, the *imd* mutant is in the Oregon R background, making the Oregon R control appropriate in this case. The other IMD pathway mutants were added at the request of the reviewers in response to the first round of reviews, and backcrossing them into the OregonR background in a reasonable amount of time was not possible. They do show qualitatively similar results to the *imd* mutant and do not alter the main conclusions of the paper, but we chose to put these mutant results in the Supplementary Material and have included a note acknowledging the differences in background.

For the case of Toll signaling, we agree the conflicting results between the *Myd88* and *spz* mutants indicate that more work needs to be done to distinguish between genetic background and mutation effects, and therefore have moved these results to the Supplementary Material and discussed them accordingly (see response to reviewer #1, point # 4 above). Importantly, this data does not affect the main conclusions of our study. We thank the reviewer for pointing this out.

3. The differeing result with spz vs. MyD88 mutants are potentially important but could also be due to poorly control genetic backgrounds and requires more work to solidfy.

We agree. See above.

4. The connection by which tolerance, phagocytosis and metabolic shift all contribute to the mechanisms to priming is unclear, although this can be left for future investigation.

We agree that this is an exciting idea for future work.

Part III – Minor Issues: Editorial and Data Presentation Modifications

Please use this section for editorial suggestions as well as relatively minor modifications of existing data that would enhance clarity.

Reviewer #1:

(No Response)

Reviewer #2:

(No Response)

Reviewer #3:

1). Supp Fig 1A: fly has gained re-infection survival from priming, some quantification would be appreciated here. HR between 1 day vs 7 days? Additionally

We have now edited Supplementary Figure 1A to include the HR's between 7 days and all other tested time intervals. The HR between a 7 day and 1 day rest between initial and secondary infection is 0.78, p = 0.26.

2). P-values for the hazard ratios are present in supp table 1, these values should be included in the text and figures/figures' legends.

P-values adjusted for multiple testing have been added to survival curves throughout figures and have been modified as appropriate in the text.

3). Why are eater flies surviving better in the PBS/PBS condition, compared to OrR flies?

Based on our reading of the literature, we don't have a clear hypothesis for why, in the PBS/PBS condition, the eater flies are surviving better than Oregon R. This may be an interesting avenue for future analyses and we have briefly noted this observation in the manuscript:

Line 419: "Our *eater* mutants also showed increased survival in response to double-wounding alone, which indicates that either the mutation or the genetic background confers increased baseline survival to repeated wounding."

4). Is is possible to track the two subsets of low-dose injected flies (the resisting and the tolerating) to see which population survive better after the high-dose 2nd challenge? (Fluorescent microbes?)

Our lab is currently developing bioluminescent bacterial strains that would allow for such an experiment. While we currently have a validated strain of bioluminescent *E. coli*, we are still working on creating a bioluminescent strain of *E. faecalis*.

5). Line 324: Does "edges" mean "lines"?

In this instance, 'edges' is referring to the lines connecting terms. We adopt this terminology to match the terminology used in the publications describing this method.

6). Line 372: "we were also identified as" – grammar mistake

Corrected in the manuscript.

7). This reviewer appreciates the authors' effort in explaining the network presentation of GSEA result. Perhaps the authors should highlight the significance of using this novel presentation method instead of using the traditional enrichment score plot?

We are glad you appreciated the GSEA description. We have added the following to the text:

Line 218: "Visualizing our GSEA results as a network of enriched terms identifies global enrichment trends in our dataset, rather than focusing on individual terms."
